# Genetic transduction by phages and chromosomal islands: The new and noncanonical

**DOI:** 10.1371/journal.ppat.1007878

**Published:** 2019-08-08

**Authors:** Yin Ning Chiang, José R. Penadés, John Chen

**Affiliations:** 1 Department of Microbiology and Immunology, Yong Loo Lin School of Medicine, National University of Singapore, Singapore; 2 Institute of Infection, Immunity and Inflammation, College of Medical, Veterinary and Life Sciences, University of Glasgow, Glasgow, United Kingdom; Nanyang Technological University, SINGAPORE

## Introduction

Bacteriophages, or phages, are the viruses of bacteria. They are obligate intracellular parasites whose existence is fatefully coupled to the success and survival of the hosts they infect and kill. Known to be the most abundant biological entities on the planet, phages are pervasive to almost all microbial communities, in which they play central roles in moderating bacterial populations and mediating horizontal gene transfer. When phages propagate, they can sometimes encapsidate host bacterial DNA to form transducing particles. Transducing particles are ostensibly like mature phage particles, only they eject bacterial DNA instead of a viral genome when they infect other cells. The DNA can then recombine into the chromosome or replicate as a plasmid in the new host cell. This process of transferring bacterial DNA from one bacterium to another is known as genetic transduction.

The genetic cargo carried in transducing particles can have very profound effects on the bacterial recipients. For example, genes that encode for antibiotic resistance or virulence factors can impart new capabilities and unlock new ecological niches, which can accelerate the emergence of new strains that are progressively more virulent and resistant to antibiotics. Although there are several mechanisms of horizontal gene transfer, phage transduction is often thought to be the major route by which bacteria acquire the genes that enable their rapid adaption to evolving environmental challenges.

Historically, all phage-mediated gene transfer was thought to occur by one of two well-described mechanisms. Since their discovery in the 1950s, generalized and specialized transduction stood as the only mechanisms of phage transduction—until now. With the recent discovery of lateral transduction, there are now three modes of phage transduction. Here, in this short review, we will provide a brief overview of what is known and what is new in phage transduction. We will also discuss noncanonical examples of transduction in which pathogenicity islands exploit phages for horizontal gene transfer.

## The phage lytic cycle and viral genome packaging

**Fig 1:**
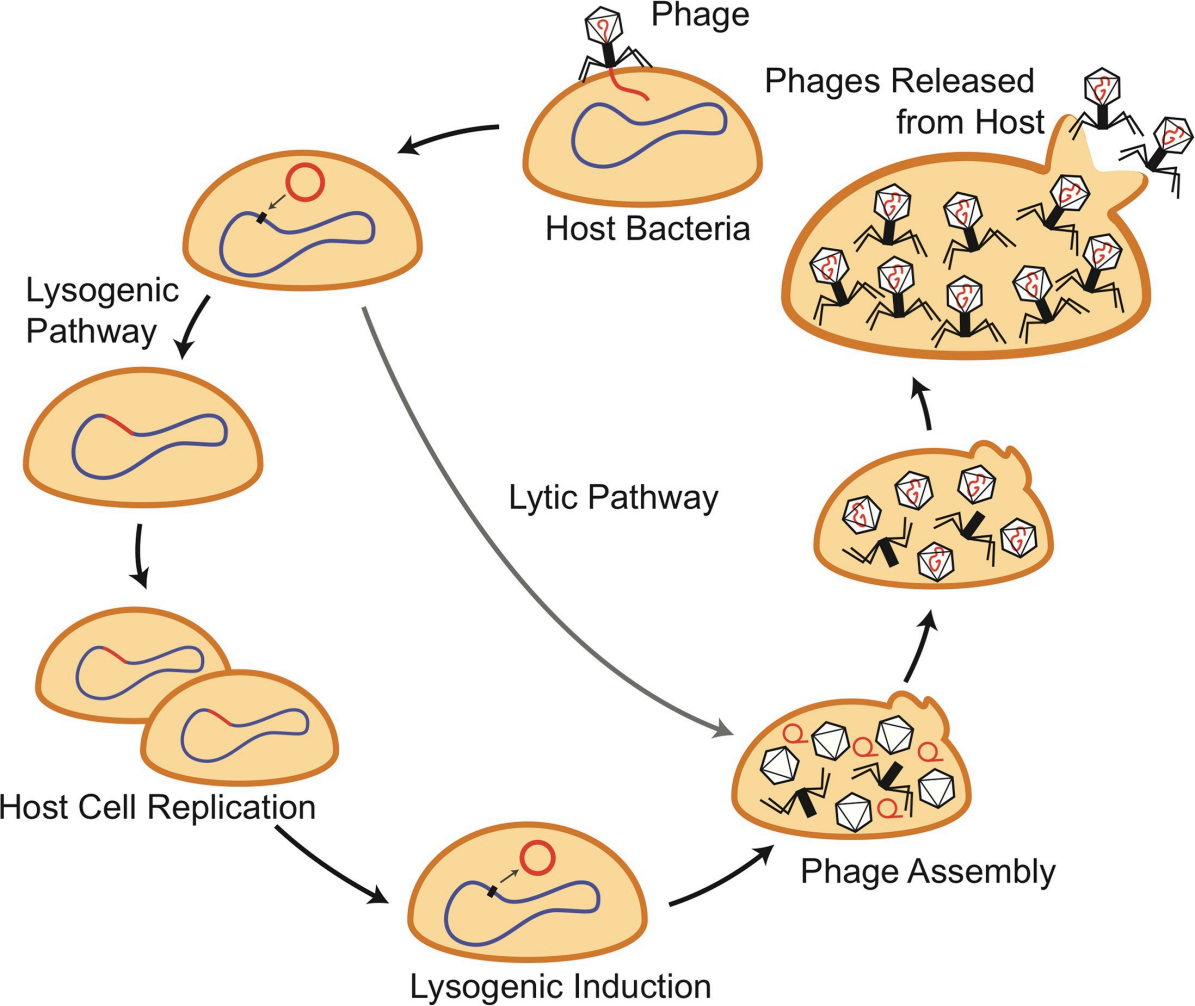
Phage life cycles. Temperate phages can insert their genomes (in red) into the host bacterial chromosome (in blue) to become a prophage in the lysogenic life cycle. Following lysogenic induction, prophages excise and enter the lytic life cycle.

Lysogenic bacteria are host cells that carry one or more prophages. Prophages are latent temperate phages that are most often stably integrated into the host bacterium’s genome, where they replicate passively as DNA during cell division. Phage development occurs in the lytic cycle following host cell infection or prophage induction from the lysogenic cycle (Fig 1). Transducing particles are also formed in the lytic cycle, and the acquisition of their content depends on the type of DNA packaging mechanism (*pac* or *cos*) utilized by the phage.

For most phages, packaging of the viral genome occurs after DNA replication. One of the first steps in the lytic cycle is the episomal circularization of the viral genome. Once circularized, the genome undergoes several rounds of theta replication, followed by a switch to rolling circle replication that produces long head-to-tail concatamers [1, 2]. Packaging of the concatameric DNA is mediated by the phage terminase enzyme (a hetero-oligomer of small and large terminase proteins), which recognizes a phage-specific packaging site (*pac* or *cos*) and cleaves the DNA to begin processing the genome into phage heads [3]. When a capsid “headful” capacity has been reached, *pac*-type terminases make a nonspecific second cut to complete packaging [4, 5]; by comparison, *cos*-type terminases require a second *cos* site to make the second cut [6]. At this stage, separation of the DNA protruding from the capsid is critical because it prevents tail attachment and maturation of the phage particle. Once the terminal cleavage is made, the highly processive terminases remain bound to the concatameric DNA and reinitiate packaging. New procapsids are filled as the *pac*-type terminases advance down the viral DNA by headfuls or as the *cos*-type terminases advance by increments of *cos* site recognition so that multiple phage heads are filled from a single concatameric genome [7].

## Generalized and specialized transduction

Generalized transduction, discovered in *Salmonella* phage P22, was the first mechanism of phage-mediated gene transfer to be identified [8]. It is the process by which phages can package any bacterial DNA (chromosomal or plasmid) and transfer it to another bacterium. The transducing particles of this mode of transduction form when bacterial host DNA is packaged into phage heads instead of viral DNA. One of the early models for how this occurs proposed that the host bacterial genome is degraded into fragments in the lytic cycle so that phage heads could package the smaller DNA pieces of suitable length. However, this theory turned out to be inaccurate because phages capable of generalized transduction are not known to cause the breakdown of the bacterial chromosome into phage-sized or smaller DNA fragments [9, 10]. Curiously, this model remains the most commonly depicted in diagrams and summary figures of generalized transduction, though it has yet to be substantiated.

**Fig 2:**
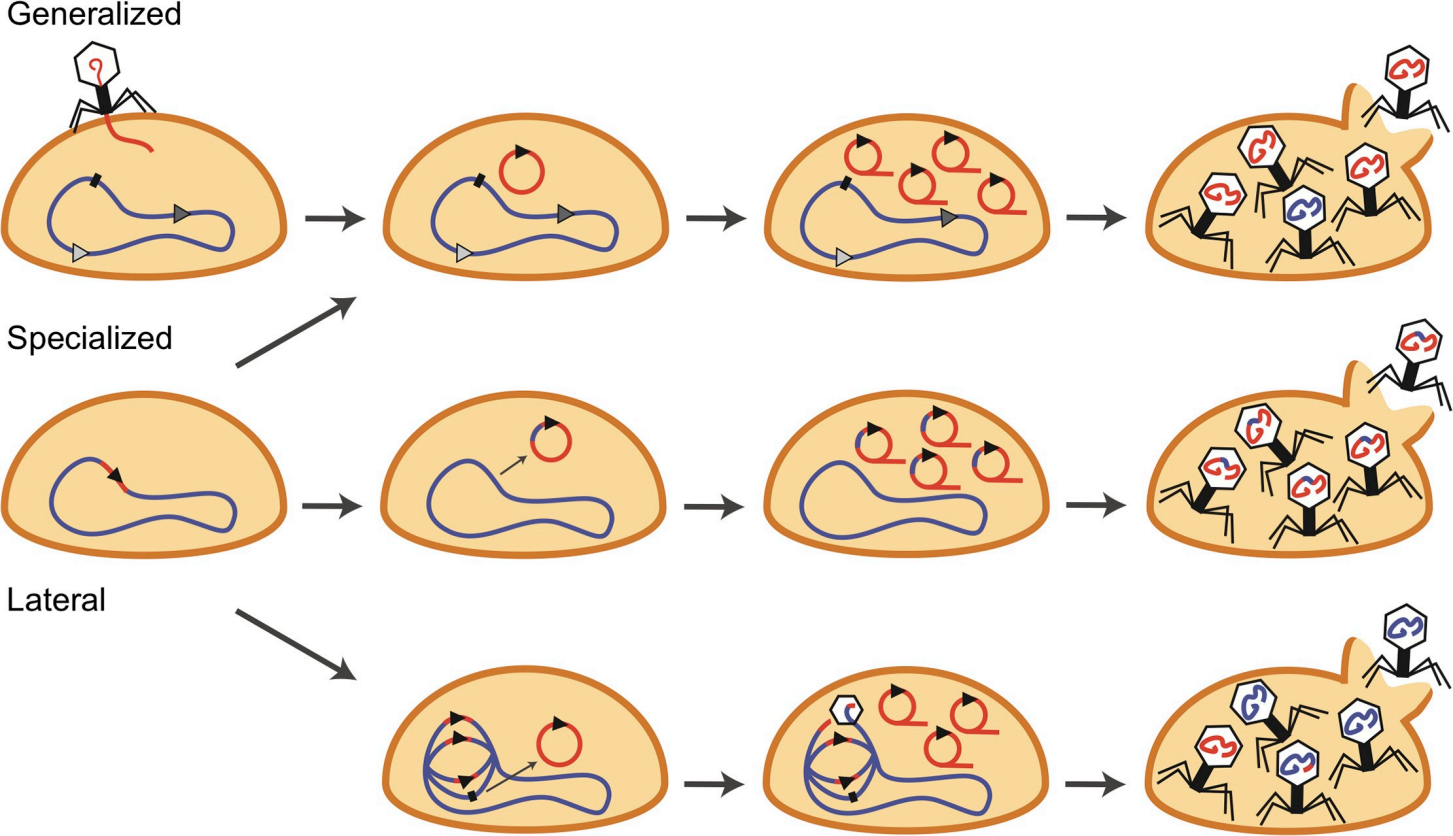
Mechanisms of genetic transduction. Generalized (top), specialized (middle), and lateral transduction (bottom). The viral genome (in red) first undergoes theta replication, followed by rolling circle replication. In lateral transduction, theta replication occurs prior to prophage excision. Phage terminase initiates DNA packaging from phage *pac* sites (black triangles) or pseudo-*pac* sites (gray triangles).

Our current understanding of generalized transduction is based on studies of *pac*-type phages such as P22, in which unfragmented chromosomal DNA serves as the substrate for packaging by the headful packaging mechanism. Here, phage terminases mistakenly recognize pseudo-*pac* sites (*pac* site homologs) in the host chromosomal or plasmid DNA and initiate packaging (Fig 2) [11–13]. Pseudo-*pac* sites vary in their degree of homology to the bona fide *pac* site and their distribution around the chromosome so that transduction frequencies vary for different parts of the genome; however, no matter the transduction frequency, the process is referred to as “generalized” because it is assumed that any part of the host genome can be packaged and transferred in this manner. For the most part, generalized transduction is exclusively mediated by *pac*-type phages because only one pseudo-*pac* site is necessary for headful packaging. Pseudo-*cos* sites also exist in the bacterial chromosome, but the chances of two *cos* site homologs occurring within a headful distance apart is extremely unlikely. Therefore, it is assumed that *cos*-type phages are not involved in generalized transduction.

Specialized transduction, discovered in coliphage λ, was the second mechanism of transduction to be identified [14]. Unlike the generalized mechanism that can package and transfer any bacterial DNA, specialized transduction is limited to the transfer of specific sets of genes. Specialized transducing particles arise when viral and bacterial host DNA are encapsidated as a hybrid molecule. The mechanism that forms these types of transducing particles is based on the classical λ phage model, in which aberrant prophage excision events join part of the viral DNA to an adjacent segment of DNA from the host bacterial chromosome in the excised molecule. Once episomal, the hybrid molecule replicates like a normal viral genome would, and if it has retained a *cos* site, the concatameric hybrid DNA can be packaged into phage heads by the *cos*-type packaging machinery and transferred to new host cells (Fig 2). Because aberrant excision is rare, and the bacterial DNA that can be transferred is restricted, specialized transduction is believed to make only a small contribution to all phage-mediated gene transfer. Interestingly, although specialized transduction is generally regarded as the domain of *cos*-type phages, it also works with *pac*-type phages [15, 16].

## Lateral transduction

The generalized and specialized modes of transduction are commonly viewed as missteps made by phages that result in the packaging of host DNA. Mistakes in *pac* site recognition are relatively rare, and errors in prophage excision even more so, and these are reflected in the low frequencies of host gene transfer normally observed for both mechanisms. Recently, the third mechanism of transduction, lateral transduction, was discovered in the temperate phages of *Staphylococcus aureus* [17]. Unlike its predecessors, lateral transduction does not appear to be the result of an erroneous phage process. On the contrary, it seems to be a natural part of the phage life cycle [18]. The key here is that the staphylococcal prophages do not follow a typical lytic program but instead excise late in their life cycle. This results in a mode of transduction that transfers bacterial chromosomal DNA at frequencies at least 1000-fold greater than previously observed.

It has normally been assumed that prophages excise and circularize early after lysogenic induction, in following a standard excision–replication–packaging pathway. The sequence in which these events occur is thought to be critical because DNA packaging prior to excision would irreversibly compromise the viral genome by cleaving it in two. Remarkably, the phages of *S*. *aureus* disrupt the excision–replication–packaging order, and they delay excision until late in their lytic program. This delay in excision has profound consequences for gene transfer because phage terminase is expressed and initiates DNA packaging in situ while the prophage is still attached to the bacterial chromosome (Fig 2). DNA packaging initiates from the bona fide *pac* site, rather than a pseudo*-pac* site like in generalized transduction, packaging part of the phage genome and continuing through the adjacent host chromosome for up to seven or more successive capsid headfuls before the transfer frequencies begin to blend into the low levels of generalized transduction. Large spans up to several hundred kilobases of the bacterial chromosome are packaged, in headful segments, and transferred at frequencies that are unprecedented for most mechanisms of gene transfer. Regions of the bacterial chromosome become “hypermobile platforms” of gene transfer, which is a novel take on the concept of mobile genetic elements that is defined by genomic coordinates, rather than by the DNA elements themselves. Finally, to offset the predictably catastrophic effects (splitting the viral genome in two) of in situ DNA packaging, in situ theta replication creates multiple integrated genomes so that both in situ DNA packaging and phage maturation can proceed in parallel. As a result, staphylococcal phages naturally produce extremely high titers of lateral transducing particles concurrently with wild-type phage production.

## Pathogenicity islands that manipulate phages for horizontal gene transfer

**Fig 3:**
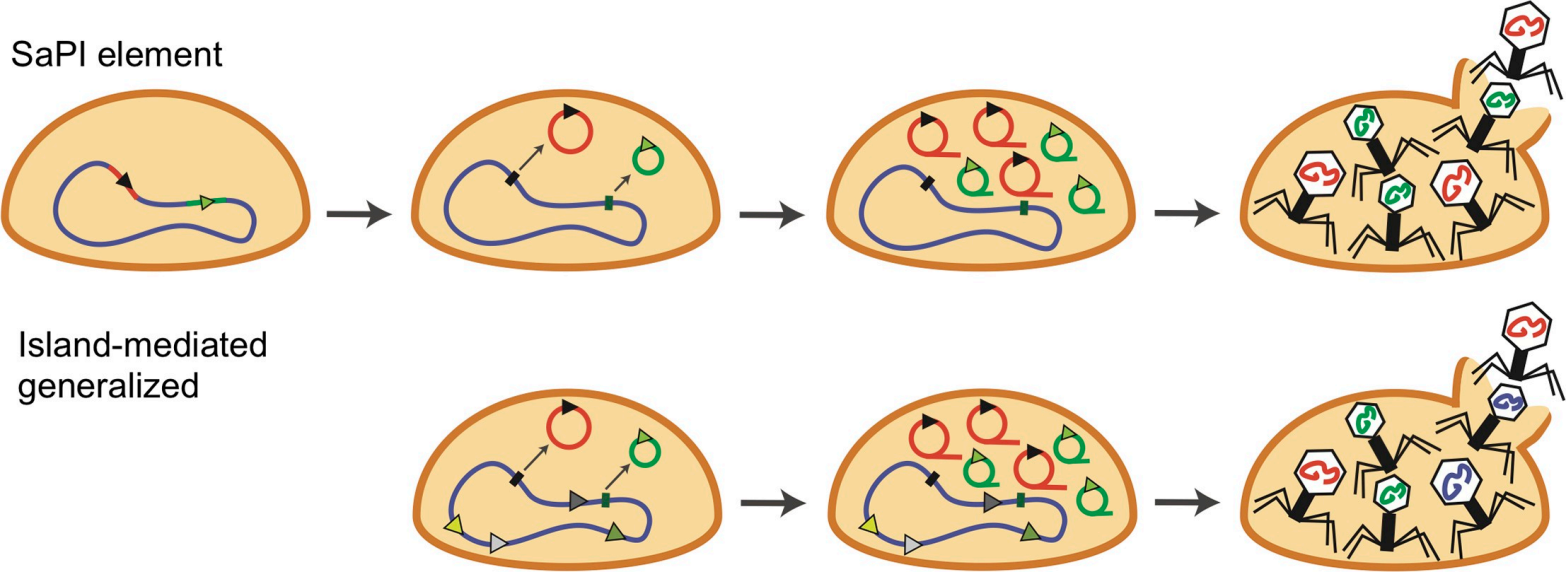
Pathogenicity island–mediated gene transfer. SaPI element transduction (top) and island-mediated generalized transduction (bottom). The strain has a viral genome (in red) and an SaPI genome (in green). Upon lysogenic induction, both phage and SaPI genomes undergo theta replication, followed by rolling circle replication. Phage terminase initiates DNA packaging from phage *pac* sites (black arrows) or phage pseudo-*pac* sites (gray arrows). SaPI terminase initiates DNA packaging from SaPI *pac* sites (green triangles) or SaPI pseudo-*pac* sites (light green triangles). SaPI, *S*. *aureus* pathogenicity island.

Phages are the most abundant gene-transfer particles in nature, and some mobile genetic elements have evolved to use that to their advantage. The *S*. *aureus* pathogenicity islands (SaPIs) are highly mobile genetic elements that carry genes for toxic shock toxin and other virulence factors [19, 20]. They are molecular parasites that exploit certain temperate phages as helpers for their own production and dissemination. Normally, they are integrated in the host bacterial chromosome until they are induced to excise and replicate by helper phage–encoded antirepressor proteins. SaPI-encoded small terminases then form hetero-oligomers with phage large terminases to form new terminase enzymes that recognize SaPI *pac* sites (instead of phage *pac* sites), enabling SaPIs to hijack the phage packaging machinery to encapsidate their own genomes into infective phage-derived particles that are transferred at extremely high frequencies, both intra- and intergenerically (Fig 3) [21, 22]. Moreover, SaPI-like elements appear to be widespread, as phage-inducible chromosomal islands (PICIs) have now been discovered in both gram-positive and gram-negative bacteria [23, 24].

The SaPIs not only mediate their own transfer but also can independently direct the transfer of unlinked bacterial DNA that contains virulence genes [25]. In a fascinating twist on genetic transduction, the SaPIs can engage in a form of gene transfer known as island-mediated generalized transduction. Much like phage pseudo-*pac* sites, there are SaPI pseudo-*pac* sites distributed throughout the bacterial genome (Fig 3). However, an important distinction is that although phage pseudo-*pac* sites appear to be located in random positions, SaPI pseudo-*pac* sites are often linked to and direct the unidirectional packaging of *S*. *aureus* genes that are associated with virulence and disease. Therefore, although they are relatively small pathogenicity islands (generally 14–16 kb), island-mediated generalized transduction links the SaPIs to a much broader repertoire of virulence determinants than they can carry themselves.

## Concluding remarks

With the rise of superbug strains that are progressively more virulent and antibiotic resistant, the importance of understanding the drivers of bacterial evolution has never been so apparent. Now, with three modes of phage transduction, we are just beginning to appreciate that genetic transduction occurs on a scale that is far greater than we ever imagined. Remarkably, phage-mediated gene transfer may be just the tip of the iceberg when it comes to genetic transduction. The PICIs are a widely distributed family of mobile genetic elements that exploit phages for their own reproduction and transfer [23, 24]. As more and more biology of the PICIs is revealed, such as mechanisms of phage parasitism or noncanonical genetic transduction, their impact on genetic transduction and bacterial evolution may prove to be profound and expansive.

In their predator–prey dynamic, phages exert a great influence over the existence of bacteria by killing and lysing them. However, the long-term survival of their host bacteria is also in the best interest of the phage, and that is best achieved by ensuring that their hosts adapt to fast-changing environments and challenges. In some instances, phages have even been reported to scavenge antibiotic resistance genes from competing neighboring cells for their host bacteria, in a process aptly termed autotransduction [26]. In that sense, phages play one of the most important roles in microbial evolution because, as agents of horizontal gene transfer and by virtue of their sheer numbers as the most abundant biological entities on the planet, they form a massive biological network that interconnects all of the genomes in the bacterial universe for the exchange of genetic material that is essential for rapid adaptation.
